# Supersweet and waxy: meeting the diverse demands for specialty maize by genome editing

**DOI:** 10.1111/pbi.13144

**Published:** 2019-05-18

**Authors:** Le Dong, Xiantao Qi, Jinjie Zhu, Changlin Liu, Xin Zhang, Beijiu Cheng, Long Mao, Chuanxiao Xie

**Affiliations:** ^1^ Institute of Crop Science Chinese Academy of Agricultural Sciences National Key Facility for Crop Gene Resources and Genetic Improvement Beijing China; ^2^ Anhui Agricultural University Hefei Anhui China

**Keywords:** CRISPR/Cas9, crop diversification, specialty maize, *SHRUNKEN2*, *GBSS* (*WX*), supersweet‐waxy compound

Maize (*Zea mays*. L) is one of the most important cereal crops that produce the starch for feedstuff, industrial feedstock and human foods. Maize also provides specialty types, such as sweet corns, waxy corns and baby corns that are welcomed by the consumers (Tracy, 2001). Sweet corns and waxy corns are derived by modifying the starch biosynthesis pathway in seed endosperms. These specialty corns have recently met increasing demands from the consumer market (Song *et al*., 2018). To this regard, the availability of necessary genotypes in the background of elite maize cultivars should significantly accelerate breeding efficiency. The recently emerged CRISPR/Cas9 technology provides the potential to achieve such goals in a much faster manner (Dong *et al*., 2018; Li *et al*., 2017; Svitashev *et al*., 2015).

One of the mechanisms underlying sweet corns is the mutation of the maize *SHRUNKEN2* (*SH2*) gene which encodes the large subunit of endosperm ADP‐glucose pyrophosphorylase (Greene and Hannah, 1998), while the cause of the waxy corn is the mutation of the *WAXY* gene (*WX*) which encodes the enzyme GRANULE BOUND STARCH SYNTHASE I (GBSS I) that is required for amylose synthesis and determines the amylose content in both endosperm and pollens (Shure *et al*., 1983). The positions of these two genes on the biochemical pathway to synthesize starch from sucrose in maize endosperm, which involves over 20 genes, are simplified in Fig. 1a (Li *et al*., 2018).

**Figure 1:**
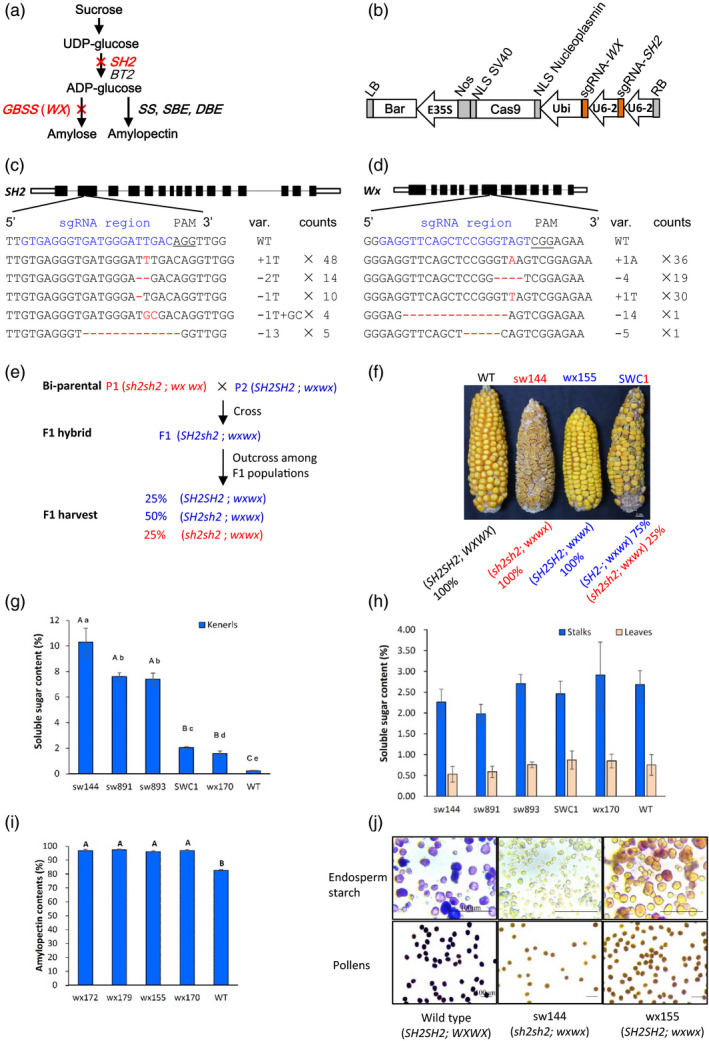
A CRISPR/Cas9‐based strategy to generate supersweet and waxy corns. (a) A simplified pathway to show the *SHRUNEN 2* gene (*SH2*) and *WX*) in maize kernels. *SH2* and *BT2* are the large (*SH2*) and small (*BT2*) subunit of ADP‐glucose Pyrophosphorylase genes; *GBSS*, the *WAXY* gene encodes the enzyme GRANULE BOUND STARCH SYNTHASE; *SS*, the soluble starch synthase genes; *SBE*, starch branching enzyme genes; *DBE*, the debranching enzyme genes; Red crosses represent CRISPR/Cas9 targeted‐knockout activity. (b) The CRISPR/Cas9 construct to target the *SH2* and *WX* genes in duplex. Bar, bialaphos resistance marker; E35S, 35S CaMV promoter; LB, T‐DNA left border; NLS, nuclear location signal sequence; Nos, Nos terminator; RB, T‐DNA right border; sgRNA‐SH2, single‐guide RNA targeting the *SH2* gene; sgRNA‐WX, single‐guide RNA targeting the *WX* gene; *Ubi*, the promoter of the ubiquitin 1 gene; *U6‐2*, the maize endogenous U6 PolIII promoter. (c) and (d) The targeted mutations on the *SH2* (c) and *WX* (d) genes. Counts, variant counts identified in 52 T1 events; Nucleotide sequence in red shows the mutations; PAM, proto‐spacer‐motif; Red dash in sequence, deletions; Blue sequence, the sgRNA location; Sequence underlined, the PAM sequence; sgRNA, single‐guide RNA; Var., variants of identified mutations; WT, the wild type. (e) and (f) The diagram of the genetic basis for SWC production (e) and the morphology of corn ears from genome edited lines (f).Texts in red indicate supersweet traits and genotypes while those in blue indicate waxy traits and genotypes. WT, ZC01; sw144, a supersweet line; wx155, a waxy line; SWC1, a supersweet and waxy line. (g) and (h) Soluble sugar contents (fresh weight%) in kernels at 22 days after pollination (22 DAP) (g), and stalks and leaves at the same time (h). A, B and C indicate statistics groups of *t*‐test at *P* < 0.01, while a, b, c, d and e represent statistics groups of *t*‐test at *P* < 0.05. Stalks of the 2nd internode below ear and leaves at ear position were used. (i) and (j) Endosperm amylopectin contents in waxy lines (i) and kernel & pollen starch phenotypes of sweet lines and waxy lines (j). (i) The endosperm amylopectin contents at mature stage. A and B, statistics groups of *t*‐test at *P *< 0.01; wx172, wx179, wx155 and wx170 are the created lines; (j) Potassium iodide (10%) assay for starch composition in kernels and pollens.

Loss‐of‐function of *SH2* (homozygous plants with a *sh2sh2* genotype) almost completely abolishes endosperm starch synthesis leading to severely shrunken and brittle seeds. These so‐called ‘supersweet’ corns accumulate more sugars and hence much sweeter (2 and 2.5 time more) than earlier versions of sweet corns such as *sugary1* that is caused by the mutation of the starch‐debranching enzyme (DBE; Pan and Nelson, 1984). Currently, supersweet corn occupies over 70% of sweet corn market due to its greater sugar content, higher kernel moisture, and longer shelf life (Tracy, 2001). On the other hand, the special chewing feeling of the endosperm texture of waxy corn (genotype *wxwx*) is preferred by many consumers. The mutation of *WX* gene shuts down the biochemical route for amylose production and allows nearly 100% amylopectin production in maize endosperm which is not only welcomed by many consumers but also useful for industrial applications (Shure *et al*., 1983). More recently, corns combining both sweet and waxy flavours have become increasingly welcomed in South‐East Asia and China and have already occupied one‐third of the Chinese specialty corm market (Song *et al*., 2018). These so‐called sweet and waxy compound corns (SWCs) are F1 hybrid seeds derived from a *sh2sh2wxwx *× *SH2SH2wxwx* parental combination, with each corn cob carrying waxy kernels and sweet kernels in a ratio of 3 to 1.

Despite the increasing economic value of specialty corns, most of them are generated using conventional maize stocks carrying genes derived from a limited number of natural mutations that were discovered several decades ago (Tracy, 2001). Introgression of recessive mutant alleles into elite modern receptor lines by backcrossing is a long and laborious process and is unavoidably subject to the undesirable linkage drag effect (Li *et al*., 2017). For SWC production, the epistasis of *SH2* over *WX* makes it more intriguing in phenotypic selection (Tracy, 2001; Li *et al*., 2018). Otherwise, a tedious marker‐assisted selection on the *wx* allele has to be employed across the backcross scheme. To this end, generation of *SH2* and *WX* mutants using more amenable technology, such as recently emerged genome editing technology, should expedite the process. Here, we adopted CRISPR/Cas9 to target *SH2* and *WX* genes in a cultivar background and identified single or double mutations that can be used to produce supersweet, waxy or SWC corns that are readily applicable in specialty corn breeding.

We developed a single construct with two sgRNAs targeting *SH2* and *WX,* respectively, (Fig. 1b) and transformed maize inbred line ZC01 using the conventional *Agrobacterium*‐mediated approach (Li *et al*., 2017). A total of 22 independent T0 transgenic plants were regenerated and characterized from ca. 800 immature embryos. Plants with a low copy number of transgenic cassettes were identified by real‐time PCR coupled with Taqman probes. Targeted regions in T0 plants were PCR‐amplified and sequenced. We found that 20 out of 22 T0 plants carried either *sh2* or *wx* alleles, or both. A total of 52 T1 plants were then derived from nine T0 plants. Targeted *SH2* and *WX* regions in T1 plants were also PCR‐amplified and sequenced, confirming that the edited sequences were inherited into the T1 plants. These genome editing‐derived small indels in T1 plants that may potentially cause frame shifts during protein translation of corresponding genes may explain the shrunken or waxy phenotypes of their seeds (Fig. 1c, d).

Interestingly, 18 out of the 52 T1 plants showed negative in bialaphos (Bar) strip tests and in PCR amplification of the CRISPR/Cas9 cassette. These plants were potential transgene‐free lines where the transgenic selection marker for the CRISPR/Cas9 cassette was removed by genetic segregation. Three ‘transgene‐free’ lines (lines 10‐24‐6, 14‐34‐6 and 17‐33‐3) were identified to be homozygous for both *sh2* and *wx* alleles (*sh2sh2wxwx*) and were named sw lines. Four lines (lines 3‐06‐4, 3‐06‐5, 8‐36‐1 and 8‐36‐2) were found to be recessive at the waxy locus only and were recorded as wx lines with *SH2SH2wxwx* genotypes. These genotypes were found to be stably inherited to the third generations (T3 plants). We then made three crosses between sw lines and wx lines and identified three potential supersweet‐waxy compound F1 plants (SWC1, 2, 3) because kernels on cobs of these lines had a ratio of 653:212 for waxy kernels to sweet kernels, fitting the expected 3:1 segregation ratio for the *SH2* locus (chi‐square test, *P* < 0.05; Fig. 1e). Samples of mature ears of sweet, waxy, SWC1 and wild‐type lines are displayed in Fig. 1f.

We then detected the soluble sugar contents in kernels on fresh ears, stalks and leaves in these specialty maize lines generated by gene editing. As shown in Fig. 1g, supersweet sw lines conferred an average of 7.38% to 10.28% sugar contents in fresh kernels, which were significantly (*P* < 0.01) higher than those in the SWC1, wx and the wild‐type lines. The sugar content in SWC kernels was significantly higher than that in wx kernels (*P* < 0.05). The fact that sugar content in wx170 was higher than that in the wild type indicates that the abolishing of the *WX* gene somehow also facilitated sucrose accumulation in wx lines. We further tested the sugar contents in stalks and leaves of generated lines and found no significant differences among sw, wx and SWC lines (Fig. 1h). This is consistent with the functional domain of the *SH2* gene which is largely associated with sugar metabolism in kernel endosperms (Greene and Hannah, 1998). On the other hand, we detected over 95% of amylopectin content in four wx lines (wx172, wx179, wx155 and wx170) using a kit based on modified concanavalin A method (Gibson *et al*., 1997), significantly (*P* < 0.01) higher than that in the wild type (82.5%; Fig. 1i). Meanwhile, potassium iodide (KI) assay showed no staining in both endosperm powder and pollens in the lines sw144 and wx155, while blue or purple colours were evident for those of the wild type (Fig. 1j), indicating nearly complete removal of amylose in kernels of wx lines. The editing of endosperm starch synthesis genes seemed no affect the other agronomic traits, which no significant difference was found between mutant lines and the wild type in plant height, days to pollinating (DTP), days to silking (DTS), kernel row number (KRN) and kernels per row (KPR; *P* < 0.01).

The availability of genome editing technology allows gene targeting in selected genetic background, overcoming the limited natural variations that severely affect breeding efficiency due to negative genetic linkage drags. We show here that genome edited materials are useful to expedite the process to produce various specialty corns with more flexibility in genetic background selection. The so‐called ‘transgene‐free’ products may be more acceptable to the public. This gene editing‐based approach also opens the door for modifying many other loci (Tracy, 2001), such as *Su1*,* Su2*,* Bt1* and *Bt2*, that could also be integrated into the scheme to produce additional specialty corns. With the total area of fresh‐table maize varieties being projected to reach 100 M ha/year in China alone (Song *et al*., 2018), the breeding of new specialty corn varieties calls for applications of new technologies to meet such demands.

## Conflict of interest

The authors declare no conflict of interest.
